# Costs and treatment patterns of incident ADHD patients - a comparative analysis before and after the initial diagnosis -

**DOI:** 10.1186/s13561-015-0078-y

**Published:** 2015-12-21

**Authors:** Mike Klora, Jan Zeidler, Roland Linder, Frank Verheyen, J.-Matthias Graf von der Schulenburg

**Affiliations:** 1 Center for Health Economics Research Hannover (CHERH), Leibniz University Hannover, Otto-Brenner-Straße 1, Hannover, 30159 Germany; 2 Scientific Institute of TK for Benefit and Efficiency in Health Care (WINEG), Hamburg, Germany

**Keywords:** Claims data, Incidence, ADHD, Costs, Germany

## Abstract

**Background and objectives:**

The costs and treatment patterns of attention deficit hyperactivity disorder (ADHD) are subjects of health services research in Germany and worldwide. Previous publications focused mainly on prevalent patients and thus research gaps were identified regarding costs and treatment patterns of incident patients before and after the first diagnosis.

**Methods:**

Analyses were conducted using claims data obtained from a large German sickness fund (Techniker Krankenkasse). Inclusion criteria consisted of patients with at least two secured outpatient or one inpatient ADHD diagnosis in 2007. Incidence was ensured by defining a baseline period without ADHD-diagnosis in 2006. In addition to diseaserelated cost analyses compared to a control group including age group comparisons, comorbidities, the proportion of multimodal treatment and medication treatment patterns were described.

**Results:**

In total, 9083 newly diagnosed ADHD patients were identified (73 % male; mean age: 12.9 years (SD: 10.3)). The mean total cost of ADHD patients during the year after the first diagnosis exceeded the mean total cost of the year before by 976 € (Differencein-Difference-estimator: 1006 €). Our analyses have shown that 10 % of ADHD patients have been treated with multimodal therapy. In addition, 11 % of the investigated ADHD population have received methylphenidate or atomoxetine preceeding the date of diagnosis in the relevant observation period.

**Discussion:**

This study provides important insights into the costs as well as the treatment patterns of incident ADHD patients. ADHD-related costs and medications can be identified prior to the date of the first ADHD diagnosis. Although, multimodal therapy is presented as an optimal treatment option by many international guidelines and experts, its proportion for treatment is low (10 %).

Further research is necessary to identify reasons for the low proportion of multimodal therapy and (cost-)effectiveness has to be evaluated in comparison to other treatment options. In addition, ADHD-related costs could be identified before the first diagnosis is documented. The reasons for medication prior to diagnosis have to be further investigated.

## Background

Attention deficit hyperactivity disorder (ADHD) is one of the most common mental disorders among children and adolescents. The global prevalence has been reported to be between 3 and 5 % [1, 2]. ADHD is defined as a dysfunctional self-controlling ability. Symptoms of the disease are characterized by an attention deficit (e.g. an inability to sustain attention on tasks or activities) accompanied with impulsivity and hyperactivity [3, 4]. Symptoms of this heterogeneous behavioral disorder commonly occur prior to the age of six [5, 6]. Furthermore, direct costs of 341 million € in 2006 demonstrate the economic burden of ADHD in Germany [7]. For ADHD treatment, a multimodal therapy consisting of medication and behavioral treatment is recommended [6]. Methylphenidate is applied as first-line medication, while atomoxetine is preferred in cases where existing comorbidities, like potential drug abuse, are diagnosed. Among ADHD patients, approximately 80 % show comorbid psychiatric disorders [6]. Therefore, comorbidities have to be taken into account, because of their impact on the appropriate choice for treatment. In addition, the cost structure for patients with comorbidities could differ from those without comorbidities.

In addition, behavioral treatment is based on occupational therapy and psychotherapy, as well as interventions in school. The aim of behavioral treatment is to improve the coping capabilities of the children. Therefore, patients need to participate in attention- and self-instruction training and coaching to sharpen their social skills [2, 8].

Existing studies in Germany focus on prevalent ADHD patients. This applies to studies focusing on economic analyses [7, 9–11], as well as publications considering treatment patterns [12]. Additionally, there is little evidence focusing on incident patients in studies from other countries. Hodgkins et al. [13] analyzed prescription and hospitalization data from the PHARMO medical record linkage database to derive incidence estimates, as well as persistence and adherence measures for the Netherlands. Ray et al. [14] analyzed costs for incident ADHD patients in the US and included the two years before and after patients’ initial diagnoses within their investigation period. One German sickness fund (Barmer GEK) conducted analyses for incident patients. However, that study includes attention deficit disorder (ADD) patients (i.e. without hyperactivity), which deviates from the current study [1]. Braun et al. [15] investigated newly diagnosed ADHD patients as well, but for subgroup analyses of treatment-persistent patients compared with a drug treatment-nonpersistent group and nondrug-treated patients. Thus, the evidence for incident ADHD patients with respect to costs and therapeutic approaches in early stages of the disease is low for Germany, and differences between international health care systems limit the ability to generalize results from one country to another. Therefore, the aim of the present study is to close this research gap and calculate the costs of treating newly diagnosed ADHD patients compared to a control group from the perspective of a major health insurance fund based on claims data as well as describing costs by age groups. Comparing costs in the investigation period before and after the initial diagnosis is another research focus. The relevance of this research question arises from the low evidence related to the amount of costs before the diagnosis compared to after the diagnosis.

In addition, there is no information whether prescriptions of methylphenidate or atomoxetine are already occurring before diagnosis. Moreover, this study will analyze whether ergotherapeutic and multimodal treatments were prescribed first, like recommended in the guidelines, or a single medication strategy was applied [6]. Furthermore, the comorbidities will be analyzed in a control group approach as well as in a pre/post comparison.

## Methods

### Data and study population

Anonymized claims data from the major German sickness fund “Techniker Krankenkasse” (TK) were available for the years 2006 to 2008. This sickness fund covered approximately 6 million insured people in 2008 who were available for this study and were studied regarding the inclusion criteria [16]. Patient identification was based on the ICD-10-GM system with ICD codes F90.0, F90.1, F90.8, and F90.9 defining ADHD. Inclusion criteria required that at least one secured outpatient diagnosis or one inpatient principal diagnosis was documented in 2007. For cases identified exclusively by outpatient diagnosis, another outpatient diagnosis within the following three quarters was necessary for inclusion. Therefore, a second diagnosis could take place in 2008, but the first diagnosis had to be coded in 2007 and was defined as index event. In order to identify patients with first-time ADHD diagnosis and to separate those patients from prevalent patients during the observation period, patients with an ADHD diagnosis in 2006 were excluded. Additionally, patients had to be continuously insured from 2006 to 2008 to be included. Since the perspective of a health insurance fund was used in this analysis, co-payments are not relevant, as they do not have a budgetary impact.

The comparison of costs before and after the first diagnosis required a follow-up period of 365 days for each individual. Scientific data validation was conducted. Besides the basic demographic information (e.g. age and gender) of the incident ADHD patients, further information on outpatient and inpatient care, drug prescriptions, prescriptions of remedies and aids as well as sick leave data were available.

### Study design

The index event is defined as the date of first diagnosis in the observation period for each ADHD patient. Since outpatient diagnoses data is only recorded as a quarter of the year in German claims data and is the most important identification source (98.4 %), the beginning of that quarter was defined as the approximate date of the index event. For instance, the index date of a patient receiving a first-time diagnosis in the second quarter of 2007 was set at 2007-04-01. ADHD patients were compared to a 1:3 matched control group adjusted for age and gender. Therefore, a direct pairwise matching took place with no replacement. The potential control group consists of all TK insured persons with no ADHD diagnosis in the whole study period. Furthermore, the index event for the control group was generated by a random function.

#### Costs

Disease- and comorbidity-related costs were identified using an incremental approach and differences by age were studied by age stratification (0-5, 6–17 and 18 years and older). The costs of the index quarter (quarter of diagnosis) were assigned the year after the index event, because a large number of ADHD-related treatments were initiated in that quarter of initial diagnosis. This method has been applied to avoid overestimation of costs, because the index diagnosis in the outpatient sector could be only identified by quarter. To avoid temporal influences and to approximate disease-related costs when compared with the control group, the difference-in-difference estimator has also been calculated. The difference-in-difference estimator compares the pre- and post-cost difference among the ADHD patients and the control group [17].

#### Comorbidities

Since the comorbidities related to ADHD could have an impact on costs, the most important comorbidities were analyzed. The odds ratio is a proven measure to verify a higher or lower chance for comorbidities in the intervention group [18, 19]. Confidence intervals were calculated using the Newcombe-Wilson method without continuity correction and the methods described by Armitage and Berry included in an Excel Tool [20–22]. Relevant comorbidities have been derived from the literature. Studies indicate that major depressive episodes and recurrent depressive disorders (F32*; F33*); mental and behavioral disorders due to psychoactive substance use (F10*-F19*); injuries, poisoning and certain other consequences from external causes (S00*-T98*); phobic and other anxiety disorders (F40*; F41*); as well as specific scholastic skill development disorders of (F81*) are often related to ADHD [5, 23]. Comorbidities have been analyzed for each patient in the year after the individual index event (0 to 364 days). For this purpose, principal diagnoses of the inpatient sector as well as secured diagnoses of the outpatient sector were included.

#### Medication and occupational therapy

Atomoxetine (ATC-Code: N06BA09) and methylphenidate (ATC-Code: N06BA04) were identified by their prescription date of service provision and reported as net values adjusted for co-payments and discounts. Occupational therapy measures were evaluated using the national remedy codes 540*, 541* and 542* in conjunction with the prescription text *Ergo*.

#### Test statistics

Data management and statistical analyses were performed with Microsoft Office Excel and Access, as well as with SPSS by IBM and SAS 9.3. Because there was no normal distribution for resource utilization and costs identified, the *U*-test following Whitney and Mann for two groups as well as the Kruskal-Wallis test for more than two groups regarding unpaired samples and the Wilcoxon rank-sum test for paired samples has been applied. Significance has been determined at the level of ≤0.05 for all tests.

## Results

### Patients’ characteristics

In total, 9083 newly diagnosed ADHD patients and 27249 control group members (1:3 matching) were identified according to the inclusion criteria. The proportion of male patients was 73 %. The gender distribution varied significantly by age (*P* = 0.000). Age at the first diagnosis averaged around 12.9 years (SD: 10.3). Male patients were diagnosed on average at 11.9 years of age, and female patients at 15.6 years of age. The distribution by age and gender (Table 1) has shown a decreasing proportion of male patients with increasing age.

**Table 1:** Patients’ characteristics

Age groups	Number of patients (control group)	Proportion of the overall amount of ADHD patients	Share of male patients in the age group
0–5	619 (1857)	6.8 %	76.9 %
6–17	7226 (21678)	79,6 %	75.4 %
18 and older	1238 (3714)	13.6 %	58.6 %
Total	9083 (27249)	100.0 %	73.3 %

### Costs

Costs of the ADHD group in the year after the index event exceeded the costs before the index event by 976 €. Compared to the control group, incremental costs added up to 720 € before the index event and 1726 € afterward, implying that the burden of disease is leading to higher costs.

The difference-in-difference (DiD) estimator of the mean total costs was 1006 €. Furthermore, it can be seen that 41 % (411€) of costs can be explained by the difference in costs for ambulatory services (Table 2). In contrast, the proportion of pharmaceuticals on total incremental costs was 13 %. Observing occupational therapy as part of the costs for remedies and aids in the ADHD group, occupational therapy was responsible for 51.4 % of costs for remedies and aids before the index event, increasing to 63.8 % afterwards.

**Table 2:** Incremental costs of the ADHD group in comparison to the control group before and after the index event (in €)

Type of costs	ADHD Group Mean [SD]		Control Group Mean [SD]		Incremental costs			difference-in-difference
	In the year before diagnosis	In the year after diagnosis	In the year before diagnosis	In the year after diagnosis	ADHD-group: After vs. before diagnosis	ADHD group to control group before diagnosis	ADHD group to control group after diagnosis	
Remedies and aids	251 [507]	367 [603]	106 [475]	104 [578]	116^a^	145^a^	263^a^	118
Outpatient care	430 [485]	801 [674]	189 [266]	149 [299]	371^a^	241^a^	652^a^	411
Outpatient surgery	7 [72]	8 [91]	6 [90]	7 [89]	1	1^a^	1	0
Inpatient care	395 [2881]	706 [3549]	157 [1734]	147 [1417]	311^a^	238^a^	559^a^	321
Pharmaceuticals	198 [1329]	332 [1245]	128 [3003]	131 [2549]	134^a^	70^a^	200^a^	130
Sick leave payments	23 [516]	45 [787]	5 [170]	13 [490]	22^a^	18^a^	32^a^	14
Rehabilitation	7 [191]	28 [397]	4 [132]	9 [301]	21^a^	7^a^	19^a^	12
Total	1311 [3502]	2287 [4179]	595 [3622]	560 [3308]	976^a^	720^a^	1726^a^	1006

^a^:significant difference (at the 0.05-α-level); *SD* standard deviation

Table 3 consists of the costs regarding the ADHD group compared to the control group stratified by different age groups. Rising total costs can be identified by age (0–5: 1898 €; 6–17: 2160 € and 18 and older: 3239 €). The same trend was identified for the incremental costs compared to the control group. In contrast to this trend observed in most service areas, the mean costs of remedies and medical aids decreased by age.

**Table 3:** Costs (€) of the ADHD group in comparison to the control group after the index event by age groups

Type of costs	0–5 years		6–17 years		18 years and older	
	ADHD	Control group	ADHD	Control Group	ADHD	Control Group
	*N =* 619	*N =* 1857	*N =* 7226	*N =* 21678	*N =* 1238	*N =* 3714
	Mean [SD]	Mean [SD]	Mean [SD]	Mean [SD]	Mean [SD]	Mean [SD]
	Median	Median	Median	Median	Median	Median
	Minimum-Maximum	Minimum-Maximum	Minimum-Maximum	Minimum-Maximum	Minimum-Maximum	Minimum-Maximum
Remedies and aids^a^	740^b^ [744]	198 [517]	368^b^ [586]	99 [606]	179^b^ [534]	86 [410]
	627	0	28	0	0	0
	0–3805	0–9056	0–8906	0–48692	0–8229	0–8598
Outpatient care	661^b^ [552]	194 [161]	796^b^ [649]	140 [311]	902^b^ [836]	179 [279]
	472	154	619	89	631	94
	61–4314	0–1407	0–8709	0–34520	0–7587	0–7451
Outpatient surgery^a^	24 [170]	16 [137]	7 [83]	6 [77]	8 [81]	13 [120]
	0	0	0	0	0	0
	0–2912	0–2879	0–2604	0–2873	0–1439	0–3153
Inpatient care^a^	323^b^ [1259]	146 [752]	641^b^ [3596]	125 [1449]	1280^b^ [3965]	269 [1478]
	0	0	0	0	0	0
	0–16932	0–20084	0–125854	0–113917	0–42933	0–39432
Pharmaceuticals^a^	133^b^ [222]	95 [337]	326^b^ [1246]	110 [1918]	465^b^ [1503]	267 [5112]
	67	45	111	21	135	14
	0–2417	0–10849	0–33929	0–224394	0–42436	0–304164
Sick leave payments^a^	-	-	-	-	330^b^ [2110]	98 [1325]
					0	0
					0–27822	0–40374
Rehabilitation^a^	17 [222]	13 [232]	22^b^ [337]	7 [247]	74^b^ [683]	24 [532]
	0	0	0	0	0	0
	0–3814	0–4944	0–12600	0–23733	0–13500	0–28659
Total^a^	1898^b^	662 [1182]	2160^b^ [4003]	487 [2834]	3239^b^ [5673]	936 [5703]
	1494	291	1355	147	1321	149
	61–21540	0–21694	25–125936	0–226983	43–54196	0–304189

^a^significant difference between the different ADHS age groups (at the 0.05-α-level); ^b^significant difference compared to the control group (at the 0.05-α-level); *SD*: standard deviation

### Comorbidities

The odds ratio for the indication of injury, poisoning, and certain other consequences of external causes for the ADHD group compared to the control group after the index date showed a range of 1.9, increasing to 19.6 for the indication of specific developmental disorders of scholastic skills. These analyses have shown a remarkable higher burden of disease for ADHD patients (Table 4).

**Table 4:** Comorbidities by age groups

Comorbidity	0–5 years			6–17 years			18 years and older			Sum		
	ADHD	Control group	Odds Ratio	ADHD	Control group	Odds Ratio	ADHD	Control group	Odds Ratio	ADHD	Control group	Odds Ratio
	*N =* 619	*N =* 1857		*N =* 7226	*N =* 21678		*N =* 1238	*N =* 3714		*N =* 9083	*N =* 27249	
	N (%)	N (%)	(95 % KI)	N (%)	N (%)	(95 % KI)	N (%)	N (%)	(95 % KI)	N (%)	N (%)	(95 % KI)
Depressive episodes/recurrent depressive disorders (F32*; F33*)	6 (1.0 %)	0 (0.0 %)	-	193 (2.7 %)	63 (0.3 %)	9.4 (7.1–12.5)	552 (44.6 %)	172 (4.6 %)	16.6 (13.7–20.0)	751 (8.3 %)	235 (0.9 %)	10.4 (8.9–12.0)
Mental and behavioral disorders due to psychoactive substance use (F10*-F19*)	0 (0.0 %)	0 (0.0 %)	-	40 (0.6 %)	29 (0.1 %)	4.2 (2.6–6.7)	151 (12.2 %)	84 (2.3 %)	6.0 (4.6–7.9)	191 (2.1 %)	109 (0.4 %)	5.3 (4.2–6.8)
Specific scholastic skill development disorders (F81*)	8 (1.3 %)	0 (0.0 %)	-	1454 (20.1 %)	268 (1.2 %)	20.1 (17.6–23.0)	41 (3.3 %)	5 (0.1 %)	25.4 (10.0–64.4)	1503 (16.5 %)	273 (1.0 %)	19.6 (17.2–22.3)
Phobic and other anxiety disorders (F40*; F41*)	11 (1.8 %)	11 (0.6 %)	3.0 (1.3–7.0)	261 (3.6 %)	181 (0.8 %)	4.5 (3.7–5.4)	249 (20.1 %)	76 (2.0 %)	12.1 (9.2–15.7)	521 (5.7 %)	268 (1.0 %)	6.1 (5.3–7.1)
Injuries, poisoning and certain other consequences from external causes (S00*-T98*)	263 (42.8 %)	512 (27.6 %)	1.9 (1.6–2.3)	2731 (37.8 %)	5481 (25.3 %)	1.8 (1.7–1.9)	422 (34.1 %)	681 (18.3 %)	2.3 (2.0–2.7)	3416 (37.6 %)	6674 (24.5 %)	1.9 (1.8–2.0)

### Treatment

Analyzing medication with methylphenidate, it became apparent that 846 (9.3 %) (atomoxetine: 62 patients; 0.7 %) patients received this agent in both periods of observation (before and after the index event). Furthermore, prescriptions of methylphenidate and atomoxetine had already occurred prior to the initial diagnosis. Thus, 3581 ADHD patients (39.4 %) (atomoxetine: 382; 4.2 %) received treatment with methylphenidate in the year following the index event, compared to 917 persons (10.1 %) (atomoxetine: 96 patients; 1.1 %) in the year before. The proportion of patients with prescriptions of both agents increased from 0.34 % before the index date to 2.2 % after the index date.

**Fig. 1:**
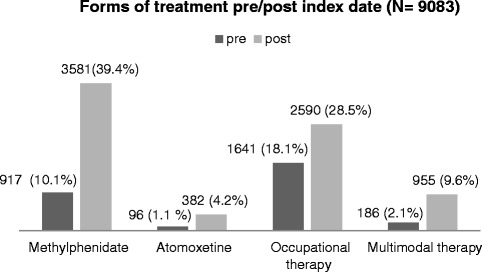
Forms of treatment pre/post index date (*N* = 9083)

Occupational therapy is an essential element in ADHD-treatment. 28.5 % of patients (2590 patients) were treated with occupational therapy after the index event (before: 18.1 % (1641 patients)). Within the first year following the index event, 9.6 % % of patients obtained multimodal therapy (Fig. 1). It should be recognized that a rise in the utilization of multimodal therapy as well as medication was identified when comparing each treatment before and after the index event. Furthermore, it has to be noticed that an increase in the use of multimodal treatment from the first to the second quarter could be identified, stagnating afterward.

There is no medication with atomoxetine or methylphenidate as well as multimodal treatment within the control group in the year before/after the index . Regarding occupational therapy 754 persons (2.8 %) were treated with this option after the index date and 902 (3.3 %) before the index date.

The mean age of those patients receiving occupational therapy after the index date was 8.3 years (SD: 4.0), which is 6.4 years lower than the mean of the remaining patients. This indicates a special application of occupational therapy for younger patients.

## Discussion

The objective of this study was to provide real-life information on costs and medication treatment patterns before and after the first diagnosis in the observation period was coded. Costs of 1006 € were identified in the control group approach (difference-in-difference). In addition, prescription prevalence for methylphenidate were calculated to be 39.4 % respectively, while prescription prevalence was lower for atomoxetine (4.2 %). This is in accordance with the German AWMF-guideline, which denotes methylphenidate as the first-line medication in ADHD [6]. It has to be emphasized that some patients were already receiving prescriptions of methylphenidate or atomoxetine in the year before the initial ADHD diagnosis.

There was a recent published study on prevalent ADHD patients. Therefore, a comparison of prevalent and incident patients is possible [24]. If a patient suffers from ADHD for a longer time, different treatment options could be used compared to incident patients. This is consistent with the fact that in the study of the prevalent ADHD patients 59 % got medication, while in this study 41 % of the incident patients were treated with medication. Congruently to the study of Braun et al. [24] identified rising mean pharmaceutical and inpatient costs and decreasing costs of remedies and aids by increasing age like in this study. But while the mean outpatient costs were decreasing by increasing age in the study of Braun et al., the outpatient costs in this paper were rising (age group: 0–5: 661 €, 6–17: 796 €, 18 years and older: 902 €) [24].

These results have also to be discussed in the context of the international existing literature, even if the comparability of country-specific results is limited due to differences in the treatment and reimbursement structure of different health care systems.

Ray at al. [14] reported the costs of ADHD patients in a control group approach for a period two years before and after the first diagnosis. The study comprised a data set from the years 1996 to 2004. The index event was defined as the first date of an ADHD diagnosis or the prescription of a relevant ADHD medication. Ray et al. [14] found incremental costs of 986 € one year after the index event (converted from $ to € with a 2007 exchange rate of 0.74 $/€). In the current study a similar amount of incremental total costs became evident (976 €) [14]. Matza et al. [25] published a review discussing the direct costs of ADHD, but did not focus exclusively on incident patients. A range of 405 € to 1081 € (converted from $ to € with an average 2004 exchange rate of 0.81 $/€) for the incremental costs provides a benchmark for the spread of costs among the matched studies. Comparing the results of Matza et al. with them of the present study, the costs are within this spread of Matza et al. [25]. Some caution has to be risen by comparing these studies. The definition of costs and the study population substantially influences the amount of total costs. Furthermore, long-term patients might already receive their optimal medication dose and have better coping mechanisms [25]. Zeidler et al. [11] examined the effects of different approaches for cost calculation in claims data studies (control group approach vs. expert approach). The authors raised the awareness of methodical transparence because of the substantial differences in calculated costs between both approaches.

Age at first diagnosis averaged around 12.9 years and did not correspond to the ICD-10 criteria, which states that the disorder should have occurred prior to six years of age [5]. A reason for the higher mean age at first diagnosis in the period of study and the low proportion of patients diagnosed prior to their sixth year of age (6.8 %) could be seen in the long period until the final diagnosis. A study proved a length of 2.6 years for this period [26]. Parr et al. [27] stated a mean age of 8.7 years at first diagnosis, while female patients were diagnosed earlier. 56 % (21 % in our study) of the female patients received their diagnoses prior to eight years of age and the proportion was 33 % among the male patients (24 %). A study of the Barmer GEK dataset analyzed the age at first diagnosis for AD(H)D patients. The male patients diagnoses were documented at a mean age of 7.5 and the females diagnoses were made at an average age of eight. Among the studies observing incident patients, this analysis is the only one based on German data. Nevertheless, embedding ADD patients is a limitation when comparing studies. The study of Gebhardt et al. [1] included patients without hyperactivity. A further study of incident patients presented an average age of 6.7 years at first diagnosis when considering children between 2 and 10 years of age [14]. The higher age of female patients at first diagnosis found in the present study is an indicator that female patients tend to be classified more often as an inattentive subtype. The inattention leads to later identification when compared with the impulsive male patients [28–31].

A large range for comorbidities can be found in the literature related to ADHD. Kessler et al. [32] computed an odds ratio for prevalent patients of 2.7 for depressive diseases, an odds ratio of 3.2 for generalized anxiety disorders, and a range of 1.5 to 7.9 for substance abuse. The current study found an odds ratio of 10.4 for depression during the follow-up period and thus, this odds ratio is higher than for the prevalent patients observed by Kessler et al. [32]. Congruently to the study of Braun et al. [24] it can be observed that there is a low proportion of patients with comorbidities in the age group 0 to 5 years except the comorbidity of injuries, poisoning and certain other consequences from external causes. The comorbidity of phobic and other anxiety disorders is increasing by age in both manuscripts. All available odds ratios by age are higher than in the study by Braun et al. except specific scholastic skill development disorders in the age group 18 and older [24]. Therefore, a higher burden of comorbidities for incident patients can be assumed.

Within the observation period, the age at first diagnosis for those who received occupational therapy was about six years lower than for those patients who did not get occupational therapy. A methylphenidate prescription for children under six is only granted under exceptional circumstances. This further strengthens the importance of behavioral treatments for this age group [6]. The multimodal therapy is predominantly stated as central treatment in the literature [33]. A comprehensive evaluation of behavioral and multimodal treatment for ADHD is urgently necessary [34].

### Limitations

This study provides important insights into the treatment patterns and the costs of incident ADHD patients in Germany. However, some limitations have to be mentioned in the context of claims data analyses. Although the TK operates nationwide, there could be some limitations regarding representativeness. As this sickness fund was established for architects, engineers and technicians and the free choice of the sickness funds is only possible since 1996, there could be a bias in terms of e.g. social status [35]. In addition, claims data are primarily collected for accounting purposes. This results in missing information for some variables, e.g. the severity of the disease. Furthermore, it must be mentioned that a one-year baseline period prior to the defined disease might be short and incidence could be overestimated. Abbas et al. [36] have published a methodological review that analyzed the effect of baseline period length on valid identification of incident cases. This study verified a 23 to 43 % higher incidence rate depending on the indication of a one-year adjustment period compared to an eight-year adjustment period, which that study defines as the gold standard [36]. Furthermore, there is some evidence supporting the assumption that ADHD was already present prior to the defined index diagnosis. For instance, suspected diagnoses and prescriptions for methylphenidate and atomoxetine occurred prior to the index event. However, in this study the first diagnosis within the investigation period was explicitly defined as an incidence criterion. Furthermore, ADHD is a chronic disease, which is supposed to be documented in regular intervals. Therefore, the limitation is solely applicable for individual cases [6].

In addition, the use of methylphenidate is approved for variant indications. Methylphenidate is licensed for the indication of narcolepsy under the trade name Ritalin [37]. However, there was only one relevant patient with ADHD and methylphenidate treatment as well as a narcolepsy diagnosis within the dataset.

Moreover, there are further limitations in the context of linking occupational therapy with diagnoses. It was barely possible to identify occupational therapy in association with psychic or motoric disorders. However, an assignment to ADHD was not feasible because of the lack of association with treatment and diagnoses in German claims data. However, a higher utilization of occupational treatment could be identified due to the control group approach as a valid measure. A comprehensive demand for evaluation is postulated in the field of occupational and multimodal treatments.

## Conclusion

In summary, ADHD patients reveal higher resource utilization compared to a control group, even prior to the diagnosis. This became recognizable by the higher costs in the year before diagnosis compared with the control group as well as by the higher usage of methylphenidate and atomoxetine. Therefore, the reasons for medication prior to diagnosis have to be further investigated. Although, multimodal therapy is presented as an optimal treatment option by many international guidelines and experts, its proportion for treatment is low (10 %). Additional research concerning the effectiveness of occupational therapy and multimodal treatments in the context of ADHD incidence has to be performed. Behavioral and multimodal treatments for ADHD as a whole need a health economic evaluation.

## Abbreviations

ADHD: Attention deficit hyperactivity disorder
ADD: Attention deficit disorder
AWMF: Arbeitsgemeinschaft der Wissenschaftlichen Medizinischen Fachgesellschaften e.V (Association of the Scientific Medical Societies in Germany)
ICD: International Statistical Classification of Diseases and Related Health Problems
SD: Standard Deviation
TK: Techniker Krankenkasse
vs.: versus
